# Modulation of mTOR Signaling in Cardiovascular Disease to Target Acute and Chronic Inflammation

**DOI:** 10.3389/fcvm.2022.907348

**Published:** 2022-06-29

**Authors:** Madlen Kaldirim, Alexander Lang, Susanne Pfeiler, Pia Fiegenbaum, Malte Kelm, Florian Bönner, Norbert Gerdes

**Affiliations:** ^1^Division of Cardiology, Pulmonology, and Vascular Medicine, Medical Faculty, University Hospital, Heinrich-Heine University, Düsseldorf, Germany; ^2^Medical Faculty, Cardiovascular Research Institute Düsseldorf (CARID), Heinrich-Heine University, Düsseldorf, Germany

**Keywords:** myocardial infarction, atherosclerosis, inflammation, metabolism, rapamycin, mTOR, anti-inflammatory treatment, cardiovascular disease

## Abstract

Inflammation is a key component in the pathogenesis of cardiovascular diseases causing a significant burden of morbidity and mortality worldwide. Recent research shows that mammalian target of rapamycin (mTOR) signaling plays an important role in the general and inflammation-driven mechanisms that underpin cardiovascular disease. mTOR kinase acts prominently in signaling pathways that govern essential cellular activities including growth, proliferation, motility, energy consumption, and survival. Since the development of drugs targeting mTOR, there is proven efficacy in terms of survival benefit in cancer and allograft rejection. This review presents current information and concepts of mTOR activity in myocardial infarction and atherosclerosis, two important instances of cardiovascular illness involving acute and chronic inflammation. In experimental models, inhibition of mTOR signaling reduces myocardial infarct size, enhances functional remodeling, and lowers the overall burden of atheroma. Aside from the well-known effects of mTOR inhibition, which are suppression of growth and general metabolic activity, mTOR also impacts on specific leukocyte subpopulations and inflammatory processes. Inflammatory cell abundance is decreased due to lower migratory capacity, decreased production of chemoattractants and cytokines, and attenuated proliferation. In contrast to the generally suppressed growth signals, anti-inflammatory cell types such as regulatory T cells and reparative macrophages are enriched and activated, promoting resolution of inflammation and tissue regeneration. Nonetheless, given its involvement in the control of major cellular pathways and the maintenance of a functional immune response, modification of this system necessitates a balanced and time-limited approach. Overall, this review will focus on the advancements, prospects, and limits of regulating mTOR signaling in cardiovascular disease.

## Introduction

Ischemic heart disease is still the leading cause of death worldwide (1). At present, risk reduction through lifestyle modifications, weight management, blood pressure control, and lipid-lowering treatment achieves just a part of the prevention goal. Furthermore, in acute myocardial infarction (AMI), rapid blood flow restoration and post-infarct treatment supported by dual antiplatelet medication, ß-blockers, and angiotensin-converting enzyme (ACE) inhibitors lead to better outcomes (2).

However, in high-income nations, impaired cardiac function following myocardial infarction is the predominant cause of heart failure development, leading to chronic impairment and a decline in quality of life due to dyspnea and decreased physical ability (3). Years of healthy life lost due to disability (YLDs) increased by 106% between 1990 and 2019 (4). As a consequence, additional preventative and therapeutic strategies are required to enhance heart function following ischemic events.

The involvement of the immune system is well recognized as a key component of cardiovascular diseases (CVDs) such as atherosclerosis, a chronic inflammatory disease, but also of myocardial infarction (5). As a result, the regulation of inflammatory processes in vascular and myocardial illness has sparked significant attention as a treatment option for CVD.

Meanwhile, mTOR inhibitors have made significant progress in their utilization to modulate the immune response in a variety of disease disorders (6). The therapeutic applicability of these inhibitors is expanding, as indicated by the rising number of studies. Thus, it is critical to assess the impact of mTOR on acute and chronic types of inflammation in the setting of CVD.

In this review, the role of mTOR in acute and chronic CVD situations will be discussed. The precise function of mTOR in these pathologies will be emphasized using myocardial infarction (MI) and atherosclerosis as two notable instances of CVD defined by pathophysiological mechanisms comprising acute and chronic inflammation, respectively. The involvement of immune cells and their subtypes will be discussed in light of mTOR inhibition as a therapeutic option for reducing the excessive immune response in CVD.

## mTOR, A Master Regulator of Metabolism

**Figure 1:**
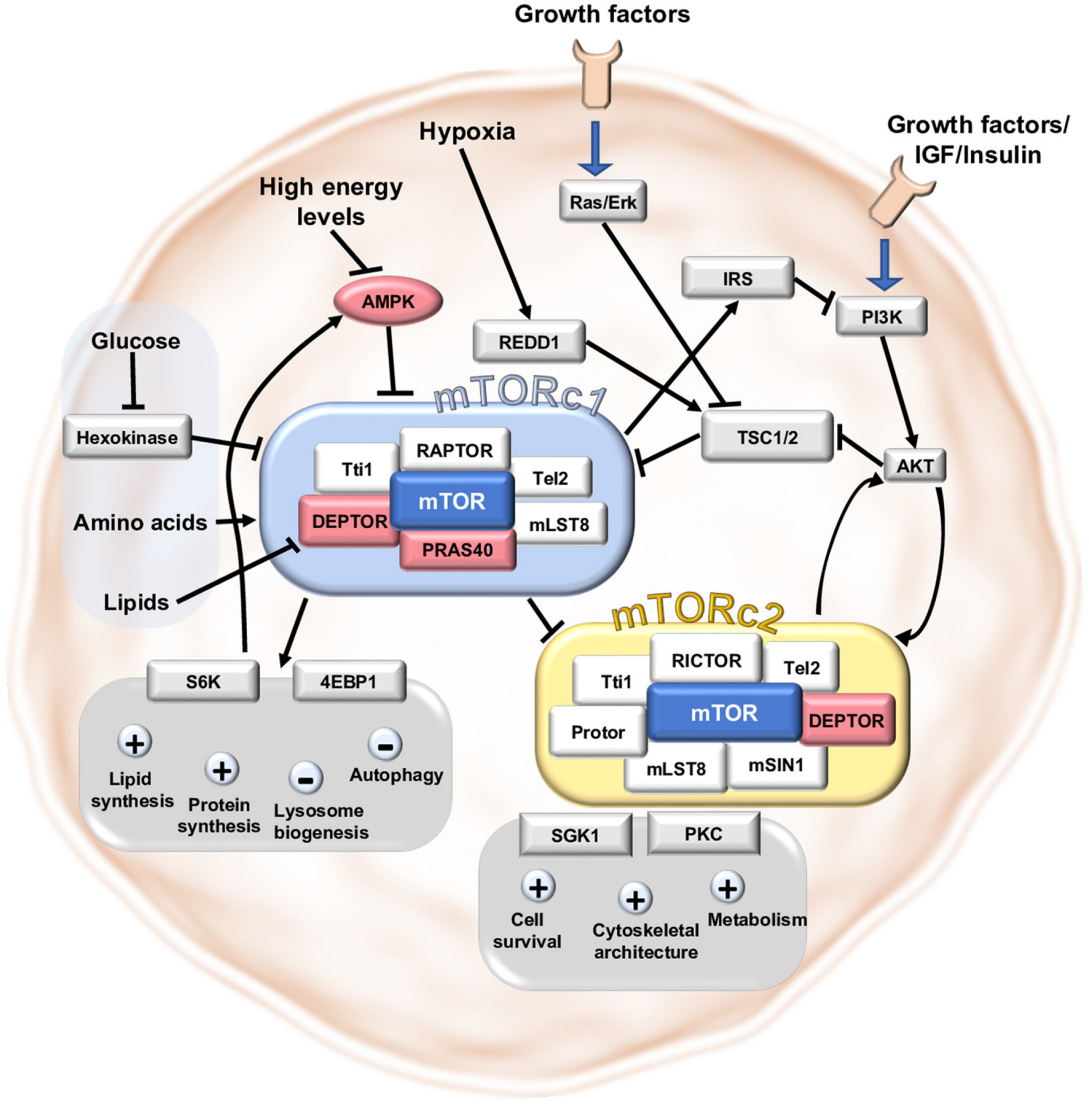
Main signaling pathways in mTORc1 and mTORc2. The mTOR signaling pathway is a central mediator between nutrient abundance and growth stimuli on the one hand and proliferation, metabolism and cell survival on the other hand. mTORc1 and mTORc2 are both complexes consisting of the protein mTOR and further necessary proteins assembling around mTOR. DEPTOR and PRAS40 impede mTORc1-activity and DEPTOR negatively affects mTORc2-activity. Upstream activation is reached after stimulation by growth signals/IGF1 or insulin via PI3K and AKT or independent of AKT via Ras/Erk-pathway. High abundance of nutrients like amino acids, glucose, lipids and high energy levels stimulate mTORc1 directly or indirectly. Hindering circumstances like hypoxia can inhibit the mTORc1 complex by stimulation of the inhibitory protein REDD1. Downstream targets of mTORc1 involve the proteins 4EBP1 and S6K to induce lipid and protein synthesis and block autophagy and lysosome biogenesis. mTORc2 can regulate cell survival, cytoskeletal architecture and metabolism by modulating SGK1 and PKC.

The mTOR signaling system is an important regulator of metabolism, cell survival, and cytoskeletal architecture (Figure 1). When the route is activated, it promotes anabolic activities like protein and lipid synthesis while inhibiting catabolic processes such as lysosome biogenesis and autophagy. The mTOR-complex itself exists as mTORc1 and mTORc2 in two distinct protein compositions. In addition to mTOR, the proteins mammalian lethal with SEC13 protein 8 (mLST8), DEP domain-containing mTOR-interacting protein (DEPTOR), TELO2 Interacting Protein 1 (Tti1), and telomere length regulation protein TEL2 homolog (Tel2) are found in both complexes (7). Regulatory-associated protein of mTOR (RAPTOR) and proline-rich Akt substrate of 40 kDa (PRAS40) are specific proteins for mTORc1 generation, whereas Rapamycin-insensitive companion of mammalian target of rapamycin (RICTOR), protein observed with Rictor-1 (Protor1), and mammalian ortholog of Stress-activated map kinase-interacting protein 1 (mSIN1) are specific proteins for mTORc2 formation (8, 9). Growth factors and insulin/Insulin-like growth factor 1 (IGF-1) operate as upstream regulators by binding to their receptor tyrosine kinase (RTK) and activating phosphoinositide 3-kinase (PI3K) and, ultimately, protein kinase B (AKT) (9, 10). The Ras/Erk/p90 ribosomal S6 kinase 1 (RSK1) signaling axis is used by alternative routes to send their signal from the RTK (7).

mTOR directly and indirectly senses nutrient levels and the cell's energy source. The lysosome is the cellular compartment where most nutrients are assembled and where the components of the mTOR-complex come together, resulting in mTOR pathway activation. By forming the complex at the lysosomal membrane with the activating protein Ras homolog enriched in brain (RHEB), amino acids induce Rag-Proteins (Ras-like small GTPase) to form heterodimers and activate mTORc1 (7, 10). Phosphatidic acid (PA) is a lipid second messenger derived from phosphatidylcholine that activates mTOR by removing the inhibitory protein DEPTOR from mTOR-complexes (10). Inactivation of mTORc1 by binding to hexokinase II can signal a shortage of glucose (11). Low energy levels in the form of low ATP abundance limit mTOR function by phosphorylating AMP-activated protein kinase (AMPK) thus activating the upstream inhibitor tuberous sclerosis complex 2 (TSC2) (12). Hypoxia interacts with mTOR via regulated in development and DNA damage responses 1 (REDD1), which also activates the upstream inhibitor TSC2 (12).

The downstream consequences of mTOR vary depending on whether mTORc1 or mTORc2 is activated. Indeed, activating mTORc1 enhances lipid and protein synthesis while inhibiting autophagy and lysosome biogenesis (7). In particular, it phosphorylates the ribosomal protein S6 Kinase-beta 1 (S6K1) which is involved in protein synthesis by promoting ribosome biogenesis (10) and inhibits AMPK, an important activator of autophagy through phosphorylation (13). This is why mTOR inhibitors are frequently used in studies of autophagy (14). mTORc1 suppresses the translation repressor 4EBP1 [eukaryotic translation initiation factor 4E (eIF4E)-binding protein 1], which can enhance anabolic pathways (10). mTORc2 primarily affects cell survival, metabolism, and cytoskeletal architecture via regulating cAMP-dependent kinases, cGMP-dependent kinases, protein kinase C, protein kinase B, and SGK1 (serum/glucocorticoid-regulated-kinase1) (10). Feedback mechanisms between mTORc1 and mTORc2 allow for reciprocal fine-tuning. mTORc2 activation of AKT results in mTORc1 upregulation, whereas mTORc1 activation inhibits mTORc2 activity via S6K1 phosphorylation. Furthermore, mTORc1 regulates its own activity by feedback regulation, which involves the suppression of IRS1 (insulin receptor substrate 1), an upstream regulator of mTORc1 function (7). mTORc2, on the other hand, can activate itself via a positive feedback loop involving AKT and stress-activated map kinase SIN1 (SAPK-interacting 1) (10).

## Immune Response in Myocardial Infarction

When the coronary blood flow is disrupted, most frequently due to coronary artery occlusion by a thrombus, the affected tissue lacks oxygen and nutrient supply and eventually enters apoptotic or necrotic programs. In this setting of sudden necrosis of cardiomyocytes damage-associated molecular patterns (DAMPs) are released (15). The activated resident immune cells increase the production of inflammatory cytokines and chemokines such as interleukin-1 (IL-1), tumor necrosis factor-α (TNFα), IL-6, and CC-chemokine ligand 2 (CCL2) lead to an increased invasion of neutrophil granulocytes and monocytes, which reinvigorate subsequent immune cell recruitment (15–18). To neutralize and opsonize necrotic cells, neutrophils release enzymes (matrix metalloproteinases (MMPs), myeloperoxidases (MPO), and elastases), complement proteins, and reactive oxygen species (ROS) (19, 20). Pro-inflammatory monocytes (CD14^+^, CD16^−^) and T cells enter the infarcted region at an early stage of the inflammatory response (15, 21–23). These early monocytes provide enzymes for tissue degradation and phagocyte debris, while also expressing additional pro-inflammatory cytokines such as interleukins, interferons (IFNs) and TNFα and present antigens on the MHC-II-complex, which promote further inflammation and involve adaptive immunity (22, 24, 25). After a few days, the necrotic cells and debris is cleared by activity of resolving macrophages (23), which also produce anti-inflammatory cytokines such as IL-10, transforming growth factor beta (TGFß), and lipid-derivates (20). Perpetuating this anti-inflammatory response, regulatory T cells (Tregs) also produce IL-10 and TGFß while regulatory macrophages secrete growth factors (e.g., VEGF) that primarily activate anti-inflammatory, angiogenic, and reparative pathways (25–27). TGFß stimulates fibroblasts to differentiate into myofibroblasts and generate persistent scar tissue, which is accompanied by a fading immune response and results in a partially recovered myocardial function (15). However, resolving inflammation and reestablishing cellular homeostasis are required for long-term healing and preventing persistent unfavorable outcomes such as heart failure.

## Immune Response in Atherosclerosis

Atherosclerosis is the underlying vascular disease that–when occurring in coronary arteries -eventually leads to AMI. It is an artery intimal layer disease that develops over extended periods of time (28). The production of atheromata is strongly linked to excessive lipid levels and other risk factors (e.g. elevated blood pressure, smoking or metabolic diseases like diabetes mellitus) (29). Endothelial dysfunction is considered the beginning point for atherosclerotic plaques, involving decreased nitric oxide levels, increased oxidative stress, and pro-inflammatory signaling (30, 31). Early inflammation in the vessels occur after exposure to high lipoprotein levels within several days (32). Lipoprotein particle uptake and phagocytosis [e.g., oxidized Low Density Lipoprotein (LDL)] triggers the formation of “fatty streaks” that develop over time toward advanced plaques composed of a necrotic lipid core covered by a fibrous cap (33). Immune cells are active at all phases of atherogenesis. The occurrence of 14 unique cell types in human atherosclerotic plaques include diverse subtypes of macrophages, dendritic cells (DCs), T cells, B cells, natural killer T (NKT) cells, neutrophils, and mast cells (34). Activated monocytes and T cells express receptors that interact with the endothelium to trigger the further activated inflammatory phenotype and guide additional inflammatory cells to the damaged area (35). By presenting antigen and excreting cytokines, macrophages and DCs in atherosclerotic lesions contribute to T cell activation. The major subpopulation of T cells present in atheromata are CD4^+^ T helper cells (36) while a minor number of cytotoxic CD8^+^ T cells predominantly localize to the fibrous cap (37). Activity of CD8^+^ T cells enables the release of enzymes that can additionally loosen the plaque structure and lead to plaque rupture worsening the prognosis in atherosclerosis (38). Recently, this concept is expanded by observations that not only plaque rupture but also superficial erosion of intimal layers of the endothelium can lead to thrombotic events and occlusion of the arterial vessel (39). Superficial erosion appears to lead through mechanical stress to a damaged and discontinuous endothelial layer that secondly involves generation of neutrophil extracellular traps (NETs) (40).

Analyzing the subgroups of the T helper cells, T helper cell type 1 (Th1 cells) represent the majority of T helper cells in atheromata and appear to be accountable for disease progression (41). Th1 cells produce IFNγ which specifically leads to enhanced lysis of collagen and reduced collagen-formation and therefore contributes to plaque instability (38). Th17 cells secrete IL-17 and foster plaque growth (42). Other cytokines like IL-6 can contribute to further foam cell accumulation (43) and TNFα further activates intercellular adhesion molecule-1 (ICAM-1), vascular cell adhesion protein (VCAM-1) and monocyte chemoattractant protein-1 (MCP-1) in the vascular wall leading to further immune cell invasion (44). Tregs, on the other hand, boost anti-inflammatory monocytes through IL-10 release, limit antigen presentation in DCs (45), and reduce overall pro-inflammatory cytokines and T cell proliferation (26, 46). Neutrophil activity inside the atherosclerotic plaque attracts monocytes to the inflammatory site and increases inflammation by cytotoxic action (47).

The immune system plays an orchestrating role in all phases of plaque formation and complication and has been the focus of numerous experimental attempts to target atherosclerosis. Several clinical trials have recently demonstrated proof of concept for the feasibility of fighting CVD by modifying the chronic inflammatory response associated with atherosclerosis (48).

## mTOR-Pathways in General and Cardiovascular-Related Immune Response

Currently, systemic mTOR inhibition is mostly utilized to treat cancer and for immunosuppression following organ transplantation. These clinical applications harness the major effects of decreased mTOR-signals: immunosuppression and decreased proliferation. Pivotal implications of mTOR-signaling are further identified for cardiovascular diseases like acute myocardial infarction, myocardial hypertrophy, atherosclerosis, and cardiomyopathies (49). Evidence for beneficial effects of mTOR-modulation in atherosclerosis comprises observation of reduced smooth muscle cell proliferation and macrophage influx as well as decreasing lipid metabolism and neo angiogenesis after mTOR inhibition (50). In daily clinical practice mTOR inhibition is used in drug-eluting stents to reduce restenosis rate (51) and after heart transplant to reduce cardiac allograft vasculopathy (51, 52).

Emerging evidence suggests that mTOR can also operate as a relevant target to alter the outcome of a myocardial infarction. Rapamycin, the prototypical mTOR inhibitor, was shown in hypertrophy experiments in mice to reduce damage following ischemia-reperfusion injury and myocardial infarction by modulating apoptosis, ERK, and NO signaling (53). Moreover, Buss et al. demonstrated that inhibiting mTOR in rats reduced infarct site, LV dimensions, and improved cardiac function (54). This observation was linked to an increase in autophagy, limited inflammation, and an overall decrease in proteasomal degradation. In the CANTOS study, reduction of inflammation was successful in reducing Major adverse cardiovascular events (MACE) in patients with atherosclerosis (48). Reduction was managed through use of a monoclonal IL-1ß-antibody (48). To date, the CLEVER-ACS clinical trial (NCT01529554) is the only study that aims to translate the idea of targeting mTOR activity in myocardial infarction into the clinical setting while laying a specific focus on the immunomodulatory function of mTOR inhibition. In this randomized prospective study patients suffering from STEMI receive either treatment with the mTOR inhibitor everolimus or placebo to evaluate beneficial effects of mTOR inhibition on myocardial inflammation affecting infarct size and myocardial function (55).

Indeed, mTOR not only promotes immune cell proliferation by increasing cell division, but it also activates immune cells and controls cell destiny by guiding immune cell maturation. In general, mTOR activation in immune cells is triggered by an excess of nutrients and growth factors, chemokines, cytokines, toll-like receptors and their ligands, which leads to increased proliferation and more accessible energy in the course of an immune response (11). The most important mTOR downstream pathways are hypoxia-inducible factor 1 (HIF1α), peroxisome proliferator-activated receptor gamma (PPARγ), sterol regulatory element-binding proteins (SREBPs), and MYC, which induce the synthesis of nucleic acids, proteins, and lipids, providing building blocks for growth, increased functional capacity, and multiplication. Elevated energy levels are maintained by a metabolic transition from mitochondrial respiration to glycolysis, which is fueled by AKT activation and inactivating phosphorylation of class IIa histone deacetylases (11, 56). This metabolic switch is observed in immune cells (57) and cardiomyocytes (58). Despite these broad effects, certain mTOR targets cause an essentially opposite response in various cell types. The effects of mTOR signaling in distinct subpopulations will be detailed in the following sections.

## T Cells

T cells primarily contribute to adaptive immune responses in inflammation by acting as cytotoxic or stimulating players for other cells (59). Of note, T cells can contribute to both pro-inflammatory and anti-inflammatory responses allowing classification into several subpopulations (60). Changes in mTOR pathway activation not only cause a proportionate rise or reduction in the proliferation and metabolic activity of all T cells, but also cause changes in the composition and size of the populations, as well as their activity and phenotype.

mTOR inhibition by active TSC1 is required to maintain naïve T cells in a quiescent state in the absence of inflammatory signals (61). In case of antigen binding via T cell receptor (TCR) and co-stimulation by the cell surface protein CD28, mTOR initiates T cell activation (61, 62). The source of energy after T lymphocyte activation switches from ß-oxidation to glycolysis, as seen in cancer cells, and is known as the “Warburg effect” (63).

Because T cell differentiation is prevalent and the effects of various subpopulations on inflammation vary greatly, crucial mTOR activity in these subpopulations is examined further. Reduced mTOR leads to an increase in the number of Tregs that have mitigating functions in atheromata and myocardial infarction as described (61, 64). The molecular mechanism behind this effect is linked to histone H3K4me2 and 3 methylation in proximity to the Foxp3 gene, which represents the lineage-determining transcription factor of this cell type (62). In general elevated Treg counts after mTOR-suppression support anti-inflammatory monocytes by IL-10 secretion (26), impair antigen presentation of DCs (45) and suppress overall pro-inflammatory cytokines (46). These processes mitigate overall inflammatory activity. In cardiac ischemia Tregs in particular lead to beneficial effects (65). The aforementioned generation of anti-inflammatory monocytes prevent an aggravation of the infarct size (66, 67). Tregs promote collagen synthesis by elevating levels of TGFβ leading to enhanced scar formation in myocardial infarction (65). The mechanism of elevated TGFß levels by Treg-activity also improves the course of the diesease in atherosclerosis by collagen formation and stabilization of the atherosclerotic plaque, too (68).

In the remaining CD4^+^ T cell compartments mTOR inhibition limits polarization and activity of Th1, Th2 and Th17 cell subsets (61, 68). Th1 and Th17 cells depend on mTORC1 whereas Th2 cells require mTORc2 activity (69). Evidence for relevant implications of these cells in cardiovascular diseases exist in atherosclerosis, where Th1 and Th17 cells emerge in the development of atherosclerosis when autoreactive CD4^+^ T cells are stimulated with ApoB peptides in an environment of inflammatory cytokines (42). The function of Th2 cells in atheromata remains indistinct but the abundance is also sparse (41). Suppression of mTOR could have a beneficial effect in impeding the development of the pro-atherogenic Th1 and Th17 cell subsets. However, recent single cell sequencing data question the separation of T helper cell-subsets that is performed to date in atherosclerotic T cells, transforming our understanding of T helper cell development in a more dynamic direction and generating questions about the implications of the mTOR pathway in the development (37).

In CD8^+^ T cells mTOR inhibition shifts the cells from an effector phenotype into a memory cell status (70). This shift weakens the implicated pro-inflammatory effects of active CD8^+^ cells, e.g. lowers IFNγ secretion and hence affects the healing process (71). In atherosclerosis, activity of CD8^+^ T cells enables the release of enzymes that can additionally destabilize the plaque structure (38), suggesting that the decrease of effector cells could be beneficial in plaque stability. In myocardial infarction, it could be assumed, that a decrease of CD8^+^ T cells by mTOR inhibition could lower apoptosis. However, deletion of CD8^+^ cells was not beneficial for the outcome after myocardial infarction (66) and even induced a rupture of the ventricle due to inadequate clearing of necrotic material (72) indicating deleterious effects of overly inhibited mTOR signaling. In the subset of NKT cells, activation is critically controlled by IL-15 using mTOR pathways. Blocking this activation impedes augmentation of glycolysis and inhibits expression of inflammatory cytokines (11, 73). Since NKT cells release granzyme B, perforin, or FasL on targeted cells, it could be presumed that the injured cells enhance the inflammatory response by secreting IFNγ (74). Limiting NKT cell activity by mTOR inhibition could beneficially limit inflammatory response and cytotoxicity in both myocardial infarction and atheroma.

Altogether, mTOR inhibition dampens the activity of pro-inflammatory Th cells and NKT cells, shifts T helper cell subpopulations in favor of Treg subpopulations with inflammation-resolving properties, and promotes conversion of cytotoxic effector T cells into a memory status. Besides a general decrease of inflammatory activity, Tregs specifically enhance a regenerative phenotype in monocytes/macrophages and block the activation of antigen-presenting cells (APC) and effector T cells (26, 45).

## Monocytes/Macrophages

Monocytes/macrophages are important cells of the innate immune system that remove debris from the site of inflammation. As described above, attraction of further immune cells as well as activation of immune cells and phagocytic function are important in both atherosclerosis and myocardial infarction.

In general, mTOR is involved in the differentiation and development of monocytes and macrophages after stimulation with granulocyte-macrophage colony-stimulating-factor (GM-CSF) (75). However, mTOR impairment does not diminish total cell numbers, demonstrating that mTOR alone does not regulate monocyte growth and differentiation entirely (76). Regardless of its overall influence on monocytes, mTOR modification can guide monocyte polarization toward either an inflammatory or anti-inflammatory phenotype. The specific pathways are still unknown, and inconsistent data on limiting mTOR activation have been obtained through diverse techniques (77). For instance an inflammatory phenotype linked to increased mTORc1 activity is observed in bone marrow-derived macrophages by TSC1 deficiency (78–80) whereas mTORc2 activity must be reduced to yield an enhanced inflammatory phenotype (77, 81). Further, modulation of the upstream activator AKT can induce contrary effects on the development of monocytes depending on the isoform of AKT (77, 82). Further regulators that influence polarization of monocytes/macrophages are upstream activators of mTOR, such as ketamine or endothelial growth factor (EGF) that enhance inflammatory monocytes/macrophages (83, 84). These observations indicate that polarization of monocytes/macrophages can be influenced by many components of the mTOR pathway with differential results, depending even on the isoform of the regulated component.

Focusing on myocardial infarction, high rates of inflammatory monocytes worsen the outcome (85). However, depletion of all monocytes with the aim to prevent tissue damage caused by inflammatory monocytes instead impairs myocardial wound healing after myocardial infarction (25) due to insufficient clearance of necrotic tissue in the absence of monocytes/macrophages. Therefore, polarization of monocytes/macrophages into a reparative phenotype could be an approach to ameliorate myocardial wound healing. As discussed above, immediate effects of mTOR modulation cause a variety of activating and polarizing consequences on monocyte/macrophage. However, mTOR inhibition could boost polarization of monocytes into a reparative phenotype through enhanced Treg generation (67).

Aside from the effect of mTOR on monocyte growth, it is also important to note that mTOR inhibition exerts relevant effects on chemoattraction by reducing the expression of MCP-1 (CCL2) (86), that is produced in various cell types like endothelial cells, smooth muscle cells (SMCs) and fibroblasts (87). This effect can be observed in both atherosclerosis and myocardial infarction. In atherosclerosis lower chemoattraction led to smaller atherosclerotic plaques with fewer macrophages (88–90).

mTOR modulation exerts various effects in monocyte development and polarization, that are not fully characterized and understood yet. Nevertheless, formation of reparative monocytes could be promoted indirectly by enhanced Treg generation after mTOR inhibition. Additionally, reduced CCL2 expression in the environment of monocytes/macrophages reduces the number of monocytes/macrophages present at the site of inflammation by limiting chemoattraction.

## Dendritic Cells

Dendritic cells are a subset of immune cells that differ from monocytes and macrophages in that they link the adaptive and innate immune systems by acting as professional APCs, i.e., presenting antigens to adaptive immune cells (91). Overall activation is mTOR-dependent and triggered by granulocyte-macrophage colony-stimulating factor (GM-CSF), FMS-like tyrosine kinase 3 ligand (FLT3L), lipopolysaccharide (LPS), and TLR (11, 92). Systemic mTOR inhibition does not only repress proliferation and activation of all DCs but results in generation of tolerogenic DCs, a subgroup occurring after stimulation with IL-4 (93). This subset of DCs is involved in the generation of Tregs and, as a result, the downregulation of the inflammatory response (11). In myocardial infarction tolerogenic DCs regulate Tregs and macrophage polarization, preserving cardiac function and promoting functional remodeling (94). Similarly, in atherosclerosis hampered DC maturation disrupts Treg formation accelerating disease progression (41). Furthermore, DC-specific TSC1 activation reduces CD8^+^ T cells (95), that enhance the risk for plaque rupture (38). However, contrary effects occur in different subsets of DCs after mTOR modulation, making it difficult to weigh up the consequences of the opposing effects. mTOR inhibitors increase pro-inflammatory cytokine expression in some myeloid DCs (mDC) and enhance antigen presentation to stimulate pro-atherosclerotic Th1 and Th17 cells (11) while suppressing inflammatory cytokines in monocyte-derived DCs (moDCs) (96).

As a conclusion, the action of mTOR inhibitors appears to be linked to the subpopulation of DCs as well as the stage of development (92). Suppression of the mTOR pathway could cause an advantage in myocardial wound healing due to enhanced amounts of tolerogenic DCs, as well as Tregs. In atherosclerosis plaque stability is rendered due to less CD8^+^ T cells. On the other hand, secretion of pro-inflammatory cytokines as well as activation of T cells can be reinforced by augmented mTOR activity. Yet, several experimental models in atherosclerosis and myocardial infarction provide evidence that mTOR inhibition in DCs lead to a beneficial outcome.

## Neutrophil Granulocytes

The mTOR pathway is involved in the metabolic stimulation of neutrophil granulocytes via GM-CSF. Activation of neutrophils increases production of inflammatory proteins such as IL-6 and cyclooxygenase 2, which contribute to the inflammatory environment (11). By mTOR inhibition, proliferation and activation of neutrophils decline and chemokine-mediated attraction of neutrophils to the site of inflammation is hampered (97, 98). Mechanistically, specific inhibition of mTORc2 causes impaired migration by reducing pseudopod formation (99).

In myocardial infarction, neutrophils are essential to decompose the ischemic area, but secreted ROS and myeloperoxidases harm viable myocardium in the border zone (20). Blocking myeloperoxidase in particular has been proven to prevent heart dilation, resulting in a better cardiac prognosis (20). Even though the reduction of neutrophil count is beneficial, a complete elimination of neutrophils should be avoided since it resulted in increased fibrosis and heart failure following myocardial infarction (100). This observation indicates once again, that a strong mTOR inhibition could be harmful, whereas a moderate mTOR suppression could weaken the inflammation.

Furthermore, active mTOR facilitates neutrophil extracellular trap (NET) formation (101, 102). In addition to the essential role of NET formation in defense against bacteria, NETs are produced in sterile inflammation as well (103). A decline of mTOR activity could attenuate pro-inflammatory signals by limited NET formation.

Little is known about mTOR modulation in neutrophils in atherosclerosis. Risk factors, such as hypercholesterinemia and hyperglycemia, already increase neutrophil production (47). Neutrophils have an impact on the onset and growth of atherosclerotic plaques (104) and high neutrophil/lymphocyte ratios worsen the prognosis in coronary artery disease (105). Therefore, mTOR inhibition could be helpful to decelerate the progression and generation of atherosclerotic plaques by overall reduction of activity and number of circulating neutrophils.

In summary, the mTOR pathway is implicated in neutrophil granulocyte activation, migration and invasion, which together lead to an enhanced inflammation. mTOR inhibition is able to diminish neutrophil activity, generation and attraction. Lower neutrophil cell counts show improved post-myocardial infarction prognosis. Since neutrophils are involved in plaque development it is likely that mTOR inhibition could reduce atherosclerotic plaque burden by lowering neutrophil cell counts.

## B Cells

B cells are lymphocytes that are primarily responsible for the humoral immune response. Other important functions include antigen presentation to T cells and further cytokine production to influence overall immune response (106).

In the early development of B cells mTOR is highly activated (107). mTOR deficiency results in overall disruption of early B cell development in conjunction with insufficient metabolic capacity (107). In later maturation stages, B cells develop into subgroups of regulatory B cells (Breg), B1 and B2 cells (108). In a mature B cell ligation of the B cell-receptor (BCR) induces an mTOR-dependent B cell activation through NFKB, MAPK and PI3K (109). IL-4 from T cells, signals through TLR, and other chemokines and cytokines (like BAFF) work in a similar way with mTOR as a downstream-target that leads to survival and proliferation (8).

Overall depletion of B cells reduces the infarct size in models of myocardial infarction (110) which could be reached by mTOR inhibition to repress B cell development. Primed B cells with damage-associated molecular patterns (DAMPs) after myocardial infarction could be decreased by mTOR inhibition so that the progression of atherosclerotic plaques fueled by primed B cells is hampered (110–112).

In atherosclerosis, mTOR-dependent B2 cells are considered pro-atherosclerotic (8, 113, 114) B1 cells and B regs also depend on mTOR-activity (115). In contrast to B2 cells, however, these cells can diminish inflammation in atherosclerosis by antibody-secretion (116) blocking the uptake of oxLDL (109) and declining the number of associated CD4^+^ T cells in case of B1 cells (117) and excretion of anti-inflammatory cytokines like IL-10, IL-35 and TGFß in B regs (118). Adoptive transfer of B1a cells, a constitutively IgM producing subgroup of B cells that expresses CD5^+^ in mice (119), was able to decrease atherosclerotic plaque size (120, 121). Considering these soothing properties of B1 cells and B regs in inflammation in atherosclerotic plaques, mTOR inhibition could harm by suppression of B1 cell and B reg generation. Apart from the described knowledge of B cell polarization specific implication of mTOR in B cell fate decision is sparse.

In summary, during acute inflammation B cell-inhibition attenuates the pro-inflammatory effect of B cells whereas in case of chronic inflammation in atherosclerosis restraining of B cells via mTOR inhibition could hamper protective antibody expression against oxLDL. Additionally, division of B cells in smaller subpopulations is emerging and knowledge about specific effects of mTOR regarding the development and polarization of these subpopulations is lacking. Information about development of B cell subpopulations under mTOR modulation could open a whole new field of interactions that need to be further investigated regarding their relevance in myocardial infarction and atherosclerosis.

## Endothelial Cells

Endothelial cells build a barrier between the blood and the inflammation site, that may lose its strict barrier function in the course of an inflammatory response. In acute myocardial injury, DAMPs and cytokines induce the expression of selectins on endothelial cells to bind leukocytes and facilitate their migration into the tissue (15). This process goes along with enhanced permeability, facilitating leukocyte transmigration (122). On the one hand, mTOR inhibition dampens cytokines preventing leukocyte recruitment due to downregulation of adhesion molecules (123). On the other hand, there is evidence that mTOR activation prevents endothelial disruption in infarction regions and preserves tight connections between cells (124). In accordance, mTOR activity is required to mediate growth and proliferation in a disrupted area in order to facilitate tissue regeneration (125). In the case of mTOR inhibition these observations could lead to limited immune cell recruitment in ECs but also to insufficient proliferation of ECs and wound healing in the necrotic area.

Mechanical stress transferred by blood flow initiates inflammatory activity and proliferation of ECs by mTOR-signaling with clinical importance in the development of atheroma. Laminar, pulsatile flow maintains a low level of proliferative and inflammatory signaling whereas turbulences and wall shear stress (e.g., at the branching point of an artery) have an pro-inflammatory and proliferative effect (126). This stimulus is conveyed via mechano-receptors involving phosphorylation of AKT and consequently activation of the mTOR pathway (127). Thus, chronic systemic mTOR inhibition could prevent emergence and growth of atheroma.

## Smooth Muscle Cells

Smooth muscle cells (SMC) contribute to plaque formation in atherosclerosis by proliferating and producing extracellular matrix (ECM). Initially, studies focused on SMCs proliferation as a major driver of atheroma development (38). Aside from anti-inflammatory benefits, Sirolimus, a mTOR inhibitor, decreased SMCs in atheroma and boosted collagen levels (128). In coronary artery disease, drug eluting stents (DES) containing antiproliferative drugs such as the mTOR inhibitor Everolimus are successfully utilized to prevent restenosis by inhibiting intimal proliferation (51). This approach delivers a high concentration of reactive chemicals to the atheroma while avoiding systemic negative effects. The supplied dose of Everolimus induces cell cycle arrest in smooth muscle cells without causing cytotoxicity (129). Everolimus-eluting stent-Implantation reduced major adverse events and the need for ischemic-driven target lesion revascularization in patients independent of indication (130).

These observations display the successful use of mTOR inhibitors in daily clinical practice to prevent further plaque growth in coronary artery disease.

## Cardiomyocytes

In ischemia necrotic cardiomyocytes release DAMPs, that set off an inflammatory cascade. Improved cardiomyocyte survival is, beyond its obvious benefit in preserving functional cells, one possible mechanism for reducing the pro-inflammatory signal. The effects of mTOR modulators on cardiomyocytes have been linked to autophagy and apoptosis. In the acute phase of ischemia activating mTOR by application of insulin reduced the number of apoptotic cells in the heart (131–133). On the other hand, in myocardial wound healing reduced mTOR signaling elevated autophagy, which improved left ventricular remodeling following myocardial infarction (134). In chronic heart failure helpful long-term effects of mTOR inhibition increased autophagy and decreased apoptosis, preventing additional cardiac functional decline (131). Generation of cardiomyocyte hypertrophy is dependent on mTOR as well (135) enabling prevention of hypertrophic remodeling by mTOR inhibition.

Apart from cardiomyocyte survival, viable cardiomyocytes in the border zone of an infarction assume an inflammatory role. After exposure to IL-1, TLR ligands, and ROS, viable cardiomyocytes contribute to cytokine and chemokine production, as well as the expression of intercellular adhesion molecule (ICAM)-1 (15). mTOR regulates the interaction between the immune system and cardiomyocytes, making it a potential target for future study.

In summary, whereas mTOR activation may protect cardiomyocytes initially after ischemia from cell death, mTOR inhibition and increased autophagy appears to have a better long-term impact in the wound healing process (136).

## Fibroblasts

After myocardial infarction, fibroblasts are primarily involved in scar formation and the resolution of the inflammatory process. TGFß is important in myofibroblast transformation, which leads to proliferation and ECM synthesis and occurs in a higher abundance after mTOR inhibition (27). For ideal remodeling, a balance between critical scar tissue growth to avoid rupture and fibrosis is required (15). In both acute and chronic remodeling, mTOR inhibition can reduce cardiac fibrosis by repressing scar development into a mild phase (134).

## Potential Future Clinical Implications

In current clinical practice, mTOR-inhibiting compounds are essential in the treatments of malignancies, transplant patients and as coating in drug-eluting stents. Expansion of the indications to myocardial infarction could dampen overshooting inflammatory activity that damages viable cardiomyocytes in the border zone by reduced overall inflammation and fostering reparative mechanisms. In the setting of acute inflammation, a short intervention by mTOR inhibition may already mitigate the aggressive inflammatory reactions and potentially preserve myocardial function. In atherosclerotic plaques systemic mTOR inhibition could expand the beneficial effects on plaques on all vessels of the body and impede additional plaque development. To reach this aim a long-term treatment would be necessary to ensure persistent prevention of disease progression.

To translate this concept into clinical practice, side effects of these compunds have to be balanced against their benefit. Relevant side effects occur but differ depending on the dosage regimen. As mTOR is an energy and nutrition sensor, a powerful mTOR inhibitor can imitate a condition of acute food scarcity and hinder early cell growth (62). Side effects of mTOR-inhibiting therapies include severe infections as well as aplasia syndromes like anemia and thrombopenia (137) and metabolic side effects such as hyperlipidemia and hyperglycemia (138). Furthermore, poor wound healing, a risk of developing malignancies and pneumonitis must be considered when determining therapeutic dosages to treat cardiovascular disorders (139, 140). Of note, the therapy duration must be considered, as extended mTOR inhibition, for example, may increase fibrosis in myocardial infarction via persistent elevated levels of pro-fibrotic cytokines (e.g., TGFß).

Within the group of patients suffering from cardiovascular disease diabetic patients are a vulnerable subgroup prone to develop atherosclerotic plaques with a 2- to 4-fold chance to suffer from cardiovascular events (141) and higher rates in morbidity and mortality after myocardial infarction (142). This subgroup of patients could particularly take advantage from mTOR-inhibiting therapy. From the pathophysiological point of view, mTOR remains constantly activated because of persistent hyperglycemia (143). Negative feedback-loops implicating IRS (insulin receptor substrate) cause the deterioration of the insulin sensitivity (144). As described above, high levels of blood glucose led to higher inflammatory activity using the mTOR pathways. Therefore, higher baseline levels of inflammation are considered as a link between diabetes and accelerated plaque development in atherosclerosis (145).

After myocardial infarction diabetic patients also show higher inflammatory activity measured by elevated markers of inflammation (CRP and IL-6) (146, 147). Within this constellation, mTOR inhibition in myocardial infarction could interrupt the vicious cycle of constant mTOR activation and accelerated immune response to ameliorate myocardial wound healing in the critical days after the incident. Treatment of atherosclerosis would supposedly necessitate a long-term mTOR inhibition, that causes hyperglycemia itself and could deteriorate the deranged metabolism of diabetic patients (148).

## Summary

Inflammation is a substantial pathophysiological mechanism in myocardial infarction and atherosclerosis, two frequent and momentous examples for acute and chronic cardiovascular diseases. In myocardial infarction, a certain degree of inflammation is essential to decompose the necrosis, although overshooting inflammation may harm viable cells in the border zones of the affected myocardium, worsening the functional outcome. In atherosclerotic plaque development, chronic inflammation is the main process that sustains plaque growth, increasing the risk for plaque rupture and occlusion of the affected vessels. Thus, strategies that modulate inflammation may soothe aggressive metabolic processes in acute conditions, can cease chronic inflammation and build a basis for future therapies. mTOR inhibitors offer an enormous potential to interfere with the immune system because of their essential roles in central pathways of cellular proliferation, growth, and survival.

**Figure 2:**
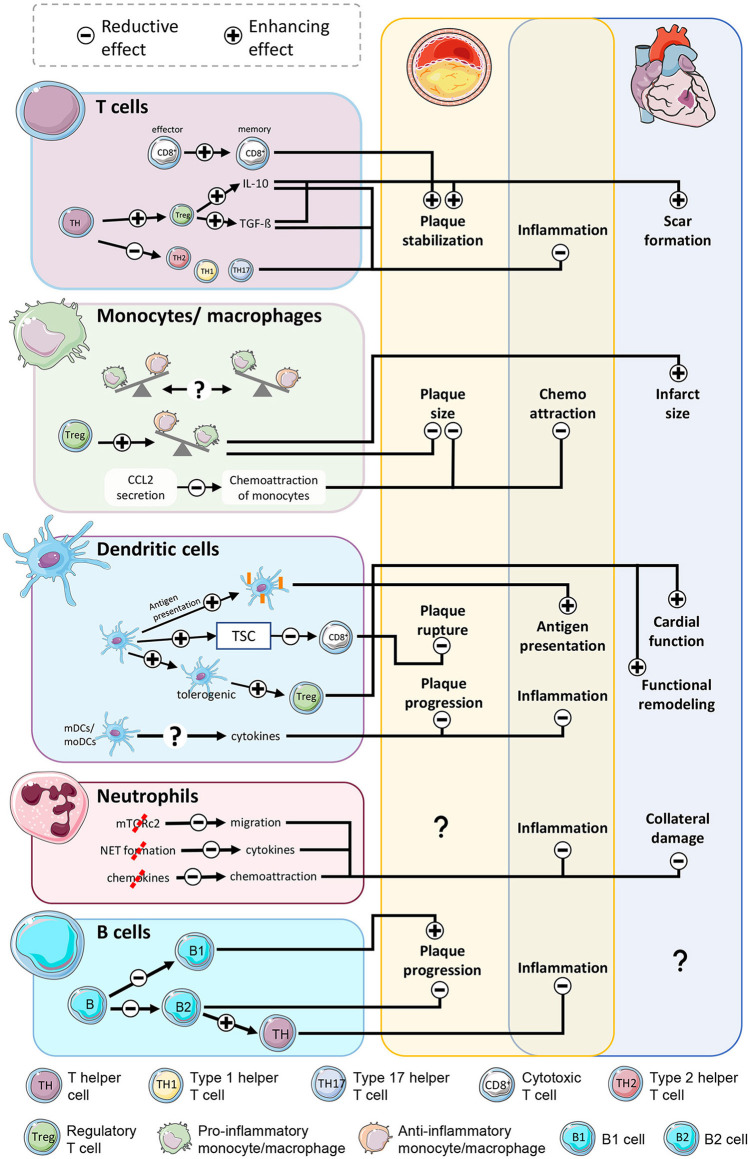
mTOR-inhibition affects atherosclerosis and myocardial infarction by modulation of immune cells. Stabilization of plaques is accomplished by less active CD8+ T cells and a shift of T helper cells to an antiinflammatory phenotype. Reduced chemoattraction reduces the number of monocytes/macrophages at the inflammation site, ameliorating inflammatory activity in both diseases. The shift of monocytes into a reparative phenotype limits the scar size. Even though enhanced antigen presentation in dendritic cells increases inflammatory activity, the shift toward tolerogenic DCs and stimulation of Treg function soothes inflammation and benefits the outcome after AMI. Reduced neutrophil activity lessens the collateral damage to viable myocardium in the border zone. B cell subpopulations exert contrary effects on atherosclerosis with yet unclarified outcome.

A large share of the beneficial effects of mTOR inhibition are based on decreased proliferation, activity and a dampening of inflammatory signals. Further, mTOR inhibition results in relevant developmental changes in specific subpopulations of immune cells relevant for the course of the disease in either atherosclerosis or myocardial infarction (Figure 2). In general, a large amount of practically every cell type is reduced, causing smaller amounts of secreted pro-inflammatory cytokines. Nonetheless, cytokines not only have a pro-inflammatory role, but they also act as messengers in a complex network with pleiotropic effects, like the anti-inflammatory cytokine IL-10. A certain number of these molecules is required to prevent negative consequences (149, 150). Chemoattraction is mostly reduced owing to lower production of inflammatory mediators, eventually leading to less-activated endothelium and reduced binding and recruitment of leukocytes. At the site of injury, this approach can already reduce numbers of neutrophils, T cells, NKT cells, B cells, monocytes/macrophages, and dendritic cells. As a consequence, clearance of necrotic/apoptotic cells is slowed, resulting in lower ROS production, degrading enzymes, and additional inflammatory cytokines, ameliorating the inflammation in both diseases. Furthermore, Tregs proliferate at a greater rate, causing monocytes and macrophages to adopt a reparative phenotype and scale down the infarct size after infarction and also stabilize the fibrotic cap of atherosclerotic plaques. Tolerogenic DCs, which are likewise boosted by mTOR inhibition may contribute to promotion of regulatory T cells and subsequently preserved cardiac function after infarction. Additionally, suppression of mTOR in mature effector cytotoxic T cells accelerated the transition of effector cells into memory cells. Reduction of the number of active cytotoxic T cells stabilizes atherosclerotic plaques. Further detailed identification of new cell types using modern methodology and ascribing these specific mTOR-regulated downstream effector function, like in B cells, will be instructive to understand the overall effect of inhibiting these pathways in CVD.

**Figure 3:**
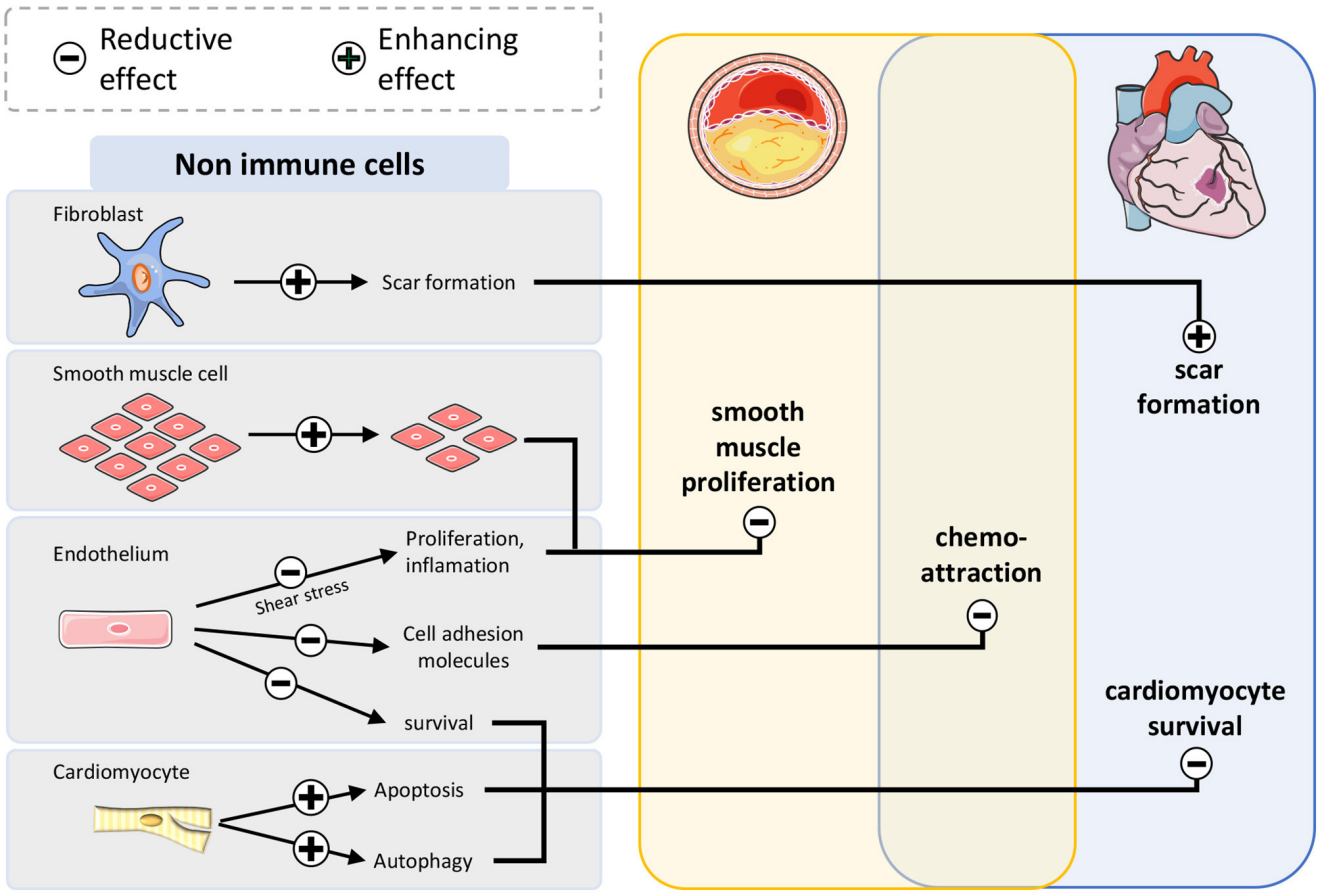
mTOR-inhibition affects atherosclerosis and myocardial infarction by modulation of non-immune cells. Smooth muscle cells proliferate less after mTOR inhibition leading to restrained plaque growth. Survival of endothelium and cardiomyocytes after infarction is impaired, leading to larger infarct size. Suppressive fibroblasts improve scar formation stabilizing the wound after myocardial infarction.

In non-immune cells (Figure 3), mTOR-inhibition disrupts survival signaling via mTOR in endothelial cells and cardiomyocytes at an early timepoint after infarction, enlarging the damaged area. In chronic inflammation mTOR inhibition prevents occlusion of coronary arteries caused by accelerated proliferation of smooth muscle cells. In fibroblasts scar formation improves when the mTOR pathway is suppressed.

To transfer mTOR inhibiting therapies into the clinical therapy of CVD further evidence is needed to titrate the correct dosing for a moderate mTOR inhibition without aplasia in all cells. Secondly, the duration and scheme of the therapy must be determined. While modulation of the immune response after infarction is needed most likely only for days to weeks after the incident, prevention of disease progression in atherosclerosis could require permanent therapy. Related to these treatment regimens the extent of side effects differs massively and ranges from high drug safety to an elevated burden in metabolic dysregulations including diabetes and hyperlipidemia, which are risk factors for atherosclerosis themselves.

In conclusion, mTOR inhibitors have a high potential to modulate inflammation in cardiovascular disorders by breaking the vicious cycle of autonomously maintained inflammation and boosting tolerance mechanisms. Even though some cells develop inflammatory properties under mTOR inhibition, anti-inflammatory and regulatory effects prevail. Apart from disease specific immune responses, this review demonstrates favorable effects of mTOR inhibition in both acute and chronic inflammation. For therapeutic usage, the dose and time range of the application must be explored further.

## Author Contributions

NG, AL, and MKa drafted the manuscript. MKa wrote the manuscript. NG, PF, and AL edited the manuscript. SP arranged the figures. Critical revision of the manuscript was made by NG, AL, PF, FB, and MKe. All authors read and approved the final manuscript.

## Funding

This study was funded by the Deutsche Forschungsgemeinschaft (DFG, German Research Foundation)-Grant No. 236177352-CRC1116; projects B06 and B09 to MKe and NG and Grant No. 397484323-CRC/TRR259; projects A05 and B03 to NG and FB. MKa is supported by a GEROK stipend of CRC1116.

## Conflict of Interest

The authors declare that the research was conducted in the absence of any commercial or financial relationships that could be construed as a potential conflict of interest. The reviewer FK is currently organizing a Research Topic with the author NG to the handling editor.

## Publisher's Note

All claims expressed in this article are solely those of the authors and do not necessarily represent those of their affiliated organizations, or those of the publisher, the editors and the reviewers. Any product that may be evaluated in this article, or claim that may be made by its manufacturer, is not guaranteed or endorsed by the publisher.
